# 2-(4,5-Di­chloro-2-nitro­phen­yl)-4-meth­oxy-3-methyl-9-phenyl­sulfon­yl-9*H*-carbazole

**DOI:** 10.1107/S1600536814001342

**Published:** 2014-01-29

**Authors:** P. Narayanan, K. Sethusankar, Velu Saravanan, Arasambattu K. Mohanakrishnan

**Affiliations:** a Department of Physics, RKM Vivekananda College (Autonomous), Chennai 600 004, India; b Department of Organic Chemistry, University of Madras, Maraimalai campus, Chennai 600 025, India

## Abstract

In the title compound, C_26_H_18_Cl_2_N_2_O_5_S, the carbazole ring system is essentially planar with a maximum deviation of 0.0498 (16) Å for the N atom. The carbazole ring system is almost orthogonal to the phenyl­sulfonyl and di­chloro-substituted nitro­phenyl rings, making dihedral angles of 84.23 (7) and 85.46 (12)°, respectively. The mol­ecular structure features intra­molecular C—H⋯O inter­actions, which generate two *S*(6) ring motifs. In the crystal, mol­ecules are linked by C—Cl⋯O halogen bonds [3.016 (3) Å, 166.63 (5)°], which generate infinite *C*(8) chains running parallel to [010].

## Related literature   

For the biological activity and uses of carbazole derivatives, see: Itoigawa *et al.* (2000 ▶); Ramsewak *et al.* (1999 ▶). For their electronic properties and applications, see: Friend *et al.* (1999 ▶); Zhang *et al.* (2004 ▶). For a related structure, see: Gopinath *et al.* (2013 ▶). For the Thorpe–Ingold effect, see: Bassindale *et al.* (1984 ▶). For bond-length data, see: Allen *et al.* (1987 ▶). For graph-set notation, see: Bernstein *et al.* (1995 ▶).
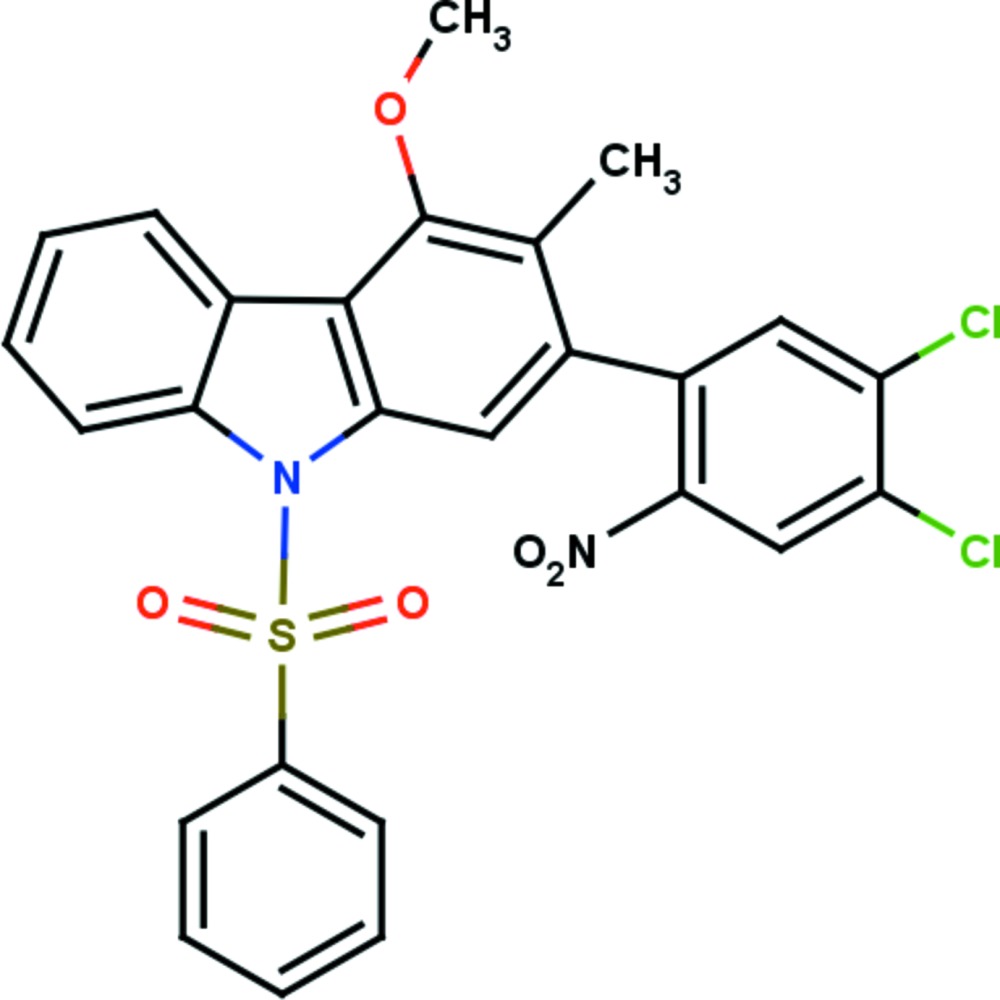


## Experimental   

### 

#### Crystal data  

C_26_H_18_Cl_2_N_2_O_5_S
*M* *_r_* = 541.39
Monoclinic, 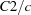

*a* = 18.6364 (7) Å
*b* = 12.1665 (4) Å
*c* = 21.1272 (7) Å
β = 91.461 (2)°
*V* = 4788.8 (3) Å^3^
*Z* = 8
Mo *K*α radiation
μ = 0.40 mm^−1^
*T* = 296 K
0.25 × 0.25 × 0.20 mm

#### Data collection  

Bruker Kappa APEXII CCD diffractometer
Absorption correction: multi-scan (*SADABS*; Bruker, 2008 ▶) *T*_min_ = 0.905, *T*_max_ = 0.923
23543 measured reflections
5218 independent reflections
4056 reflections with *I* > 2σ(*I*)
*R*_int_ = 0.031

#### Refinement  

*R*[*F* ^2^ > 2σ(*F* ^2^)] = 0.038
*wR*(*F* ^2^) = 0.112
*S* = 1.03
5218 reflections
327 parameters
H-atom parameters constrained
Δρ_max_ = 0.35 e Å^−3^
Δρ_min_ = −0.39 e Å^−3^

### 

Data collection: *APEX2* (Bruker, 2008 ▶); cell refinement: *SAINT* (Bruker, 2008 ▶); data reduction: *SAINT*; program(s) used to solve structure: *SHELXS97* (Sheldrick, 2008 ▶); program(s) used to refine structure: *SHELXL97* (Sheldrick, 2008 ▶); molecular graphics: *ORTEP-3 for Windows* (Farrugia, 2012 ▶) and *Mercury* (Macrae *et al.*, 2008 ▶); software used to prepare material for publication: *SHELXL97* and *PLATON* (Spek, 2009 ▶).

## Supplementary Material

Crystal structure: contains datablock(s) global, I. DOI: 10.1107/S1600536814001342/rk2420sup1.cif

Structure factors: contains datablock(s) I. DOI: 10.1107/S1600536814001342/rk2420Isup2.hkl

Supporting information file. DOI: 10.1107/S1600536814001342/rk2420Isup3.cml

CCDC reference:

Additional supporting information:  crystallographic information; 3D view; checkCIF report

## Figures and Tables

**Table 1:** Hydrogen-bond geometry (Å, °)

*D*—H⋯*A*	*D*—H	H⋯*A*	*D*⋯*A*	*D*—H⋯*A*
C2—H2⋯O2	0.93	2.33	2.915 (3)	121
C11—H11⋯O1	0.93	2.36	2.955 (2)	122

**Fig. 1.**
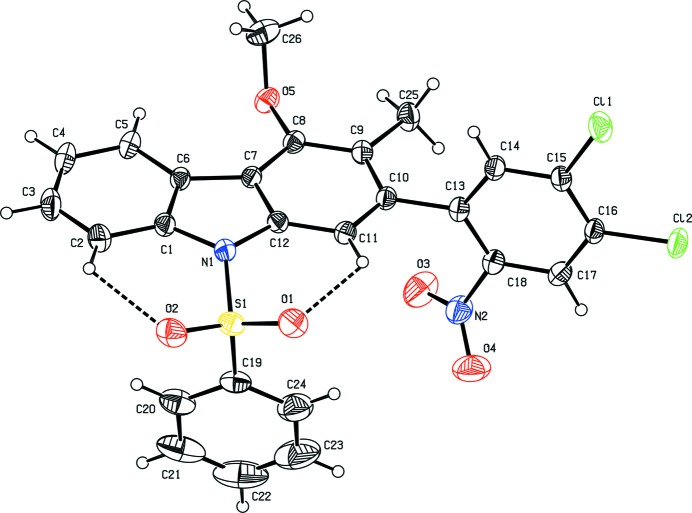
The molecular structure of the title compound with the atom numbering scheme, displacement ellipsoids are drawn at 30% probability level. H atoms are present as small spheres of arbitary radius. The intramolecular C—H···O hydrogen bonds, which are generate S(6) ring motifs, shown as a dashed lines (see Table 1 for details).

**Fig. 2.**
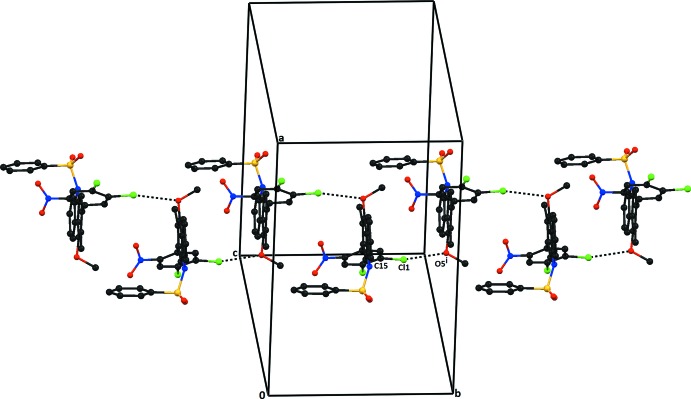
The packing arrangement of the title compound viewed down a axis. The dashed lines indicate C—Cl···O intermolecular halogen bondings. Symmetry code: (i) 1/2-x, 1/2+y, 3/2-z.

## supplementary crystallographic information

### 1. Comment

Carbazole and its derivative have become quite attractive compounds owing to their applications in pharmacy and molecular electronics. It has been reported that carbazole derivatives exhibit various biological activities such as antitumor (Itoigawa *et al.*, 2000), anti-inflammatory and antimutagenic (Ramsewak *et al.*, 1999). Carbazole derivatives also exhibit electroactivity and luminenscence and are considered to be potential candidates for electronic applications such as colour displays, organic semiconductors, laser and solar cells (Friend *et al.*, 1999; Zhang *et al.*, 2004).

The title compound, C_26_H_18_Cl_2_N_2_O_5_S, comprises a carbazole ring system which is attached to a phenylsulfonyl ring, a dichloro substituted nitrophenyl ring, a methoxy group and a methyl group. The carbazole ring system is essentially planar with maximum deviation of 0.0498 (16)Å for the nitrogen atom (N1). The methyl group carbon atom (C25) deviates from the carbazole ring by -0.0866 (22)Å. The carbazole ring system is almost orthogonal to phenyl ring attached to sulfonyl group and nitrophenyl ring with dihedral angles of 84.23 (7)° and 85.46 (12)°, respectively.

The atom S1 has a distorted tetrahedral configuration. The widening of angle O2—S1—O1 [120.21 (10)°] and narrowing of angle N1—S1—C19 [105.23 (9)°] from the ideal tetrahedral value are attributed to the Thorpe–Ingold effect (Bassindale *et al.*, 1984). As a result of electron-withdrawing character of the phenylsulfonyl group, the bond lengths N1—C1 = 1.426 (2)Å and N1—C12 = 1.418 (2)Å in the molecule are longer than the mean value of 1.355 (14)Å (Allen *et al.* 1987). The sum of the bond angles around N1 [353.9°] indicate the sp^2^ hybridization. The chlorine atoms Cl1 & Cl2 are significantly deviated by 0.1037 (6)Å and -0.0586 (5)Å, respectively from the phenyl ring (C13–C18).

The molecular structure is stabilized by C2—H2···O2, C11—H11···O1 intramolecular interactions, which generate two S(6) ring motifs (Fig. 1). In the crystal packing, molecules are linked by C15—Cl1···O5^i^ intermolecular halogen bonding (*XB*), between the chlorine atom (Cl1) and methoxy group oxygen atom (O5) of the carbazole ring system [Cl1···O5^i^ = 3.016 (3)Å and C15—Cl1···O5^i^ angle of 166.63 (2)°], which generate *C*(8) infinite one dimensional chain running parallel to base vector [0 1 0] (Bernstein *et al.*, 1995). The packing view of the title compound is shown in Fig. 2. Symmetry code: (i) 1/2-*x*, 1/2+*y*, 3/2-*z*.

### 2. Experimental

A mixture of (*E*)-1-(2-(4,5-dichloro-2-nitrostyryl)-1-(phenylsulfonyl)- 1*H*-indol-3-yl)-2-(phenylsulfonyl)propan-1-one (4.0 g, 6 mmol), dimethylsulfate (2.86 ml, 30 mmol) and K_2_CO_3_ (8.28 g, 60 mmol) in tetrahydrofuran (100 ml) was stirred at room temperature for 18 h. After completion of the reaction (monitored by *TLC*), it was poured into crushed ice (100 g). The solid obtained was filtered and dried (CaCl_2_) to give enol ether. Then, the crued enol ether was dissolved in xylenes (100 ml) and refluxed for 24 h. Removal of xylenes *in vacuo* followed by column chromatographic purification (silica gel; hexane–ethyl acetate, 8:2) gave 9-(phenylsulfonyl)-2-(4,5-dichloro-2-nitrophenyl)-4-methoxy-3-methyl- 9*H*-carbazole (2.17 g, 67%) as a colourless solid. Single crystal suitable for X–ray diffraction were prepared by slow evaporation of a solution of the title compound in chloroform (CHCl_3_) at room temperature. M.p. 493–495 K.

### 3. Refinement

The positions of hydrogen atoms were localized from the difference electron density maps and their distances were geometrically constrained. The hydrogen atoms bound to the C atoms are treated as riding atoms, with d(C—H) = 0.93Å and *U*_iso_(H) = 1.2*U*_eq_(C) for aromatic, d(C—H) = 0.96Å and *U*_iso_(H) = 1.5*U*_eq_(C) for methyl groups. The rotation angles for methyl groups were optimized by least squares.

### Crystal data

**Table:**

C_26_H_18_Cl_2_N_2_O_5_S	*F*(000) = 2224
*M**_r_* = 541.39	*D*_x_ = 1.502 Mg m^−^^3^
Monoclinic, *C*2/*c*	Melting point = 493–495 K
Hall symbol: -C 2yc	Mo *K*α radiation, λ = 0.71073 Å
*a* = 18.6364 (7) Å	Cell parameters from 4058 reflections
*b* = 12.1665 (4) Å	θ = 2.0–27.0°
*c* = 21.1272 (7) Å	µ = 0.40 mm^−^^1^
β = 91.461 (2)°	*T* = 296 K
*V* = 4788.8 (3) Å^3^	Block, colourless
*Z* = 8	0.25 × 0.25 × 0.20 mm

### Data collection

**Table:**

Bruker Kappa APEXII CCD diffractometer	5218 independent reflections
Radiation source: fine-focus sealed tube	4056 reflections with *I* > 2σ(*I*)
Graphite monochromator	*R*_int_ = 0.031
ω– and φ–scans	θ_max_ = 27.0°, θ_min_ = 2.2°
Absorption correction: multi-scan (*SADABS*; Bruker, 2008)	*h* = −23→23
*T*_min_ = 0.905, *T*_max_ = 0.923	*k* = −15→15
23543 measured reflections	*l* = −26→26

### Refinement

**Table:**

Refinement on *F*^2^	Primary atom site location: structure-invariant direct methods
Least-squares matrix: full	Secondary atom site location: difference Fourier map
*R*[*F*^2^ > 2σ(*F*^2^)] = 0.038	Hydrogen site location: inferred from neighbouring sites
*wR*(*F*^2^) = 0.112	H-atom parameters constrained
*S* = 1.03	*w* = 1/[σ^2^(*F*_o_^2^) + (0.0583*P*)^2^ + 2.8891*P*] where *P* = (*F*_o_^2^ + 2*F*_c_^2^)/3
5218 reflections	(Δ/σ)_max_ = 0.001
327 parameters	Δρ_max_ = 0.35 e Å^−^^3^
0 restraints	Δρ_min_ = −0.39 e Å^−^^3^

### Special details

**Table:**

Geometry. All s.u.'s (except the s.u. in the dihedral angle between two l.s. planes) are estimated using the full covariance matrix. The cell s.u.'s are taken into account individually in the estimation of s.u.'s in distances, angles and torsion angles; correlations between s.u.'s in cell parameters are only used when they are defined by crystal symmetry. An approximate (isotropic) treatment of cell s.u.'s is used for estimating s.u.'s involving l.s. planes.
Refinement. Refinement of *F*^2^ against ALL reflections. The weighted *R*-factor *wR* and goodness of fit *S* are based on *F*^2^, conventional *R*-factors *R* are based on *F*, with *F* set to zero for negative *F*^2^. The threshold expression of *F*^2^ > σ(*F*^2^) is used only for calculating *R*-factors(gt) *etc*. and is not relevant to the choice of reflections for refinement. *R*-factors based on *F*^2^ are statistically about twice as large as those based on *F*, and *R*-factors based on ALL data will be even larger.

### Fractional atomic coordinates and isotropic or equivalent isotropic displacement parameters (Å^2^)

**Table:**

	*x*	*y*	*z*	*U*_iso_*/*U*_eq_	
C1	0.30233 (10)	0.60315 (14)	0.46304 (8)	0.0376 (4)	
C2	0.31928 (12)	0.59976 (18)	0.39942 (9)	0.0508 (5)	
H2	0.2838	0.6021	0.3677	0.061*	
C3	0.39066 (13)	0.59284 (19)	0.38525 (10)	0.0573 (6)	
H3	0.4035	0.5891	0.3430	0.069*	
C4	0.44365 (12)	0.59128 (18)	0.43162 (11)	0.0539 (5)	
H4	0.4915	0.5875	0.4202	0.065*	
C5	0.42723 (10)	0.59515 (16)	0.49463 (9)	0.0434 (4)	
H5	0.4633	0.5941	0.5259	0.052*	
C6	0.35545 (9)	0.60060 (13)	0.51051 (8)	0.0338 (4)	
C7	0.32037 (9)	0.60416 (13)	0.57063 (8)	0.0321 (3)	
C8	0.34576 (9)	0.60187 (14)	0.63289 (8)	0.0347 (4)	
C9	0.29861 (9)	0.60208 (14)	0.68281 (8)	0.0360 (4)	
C10	0.22473 (9)	0.60638 (14)	0.66796 (8)	0.0353 (4)	
C11	0.19794 (9)	0.61140 (15)	0.60641 (9)	0.0375 (4)	
H11	0.1489	0.6162	0.5978	0.045*	
C12	0.24651 (9)	0.60903 (13)	0.55824 (8)	0.0333 (4)	
C13	0.17149 (9)	0.61156 (15)	0.71942 (8)	0.0367 (4)	
C14	0.15400 (10)	0.71305 (15)	0.74444 (8)	0.0398 (4)	
H14	0.1787	0.7750	0.7313	0.048*	
C15	0.10101 (10)	0.72501 (15)	0.78834 (8)	0.0393 (4)	
C16	0.06477 (9)	0.63314 (16)	0.81004 (8)	0.0384 (4)	
C17	0.07991 (9)	0.53179 (16)	0.78529 (9)	0.0408 (4)	
H17	0.0550	0.4699	0.7983	0.049*	
C18	0.13256 (10)	0.52280 (15)	0.74091 (9)	0.0388 (4)	
C19	0.15457 (11)	0.42952 (17)	0.46831 (10)	0.0504 (5)	
C20	0.19244 (13)	0.36252 (19)	0.42797 (13)	0.0632 (6)	
H20	0.2196	0.3928	0.3960	0.076*	
C21	0.18912 (18)	0.2500 (2)	0.43609 (19)	0.0933 (11)	
H21	0.2144	0.2038	0.4095	0.112*	
C22	0.1490 (2)	0.2064 (3)	0.4828 (2)	0.1137 (15)	
H22	0.1465	0.1305	0.4876	0.136*	
C23	0.1125 (2)	0.2727 (3)	0.5224 (2)	0.1109 (13)	
H23	0.0859	0.2421	0.5546	0.133*	
C24	0.11478 (17)	0.3856 (2)	0.51522 (14)	0.0790 (8)	
H24	0.0894	0.4310	0.5421	0.095*	
C25	0.32572 (11)	0.59430 (18)	0.75030 (9)	0.0488 (5)	
H25A	0.3757	0.5753	0.7510	0.073*	
H25B	0.2992	0.5388	0.7720	0.073*	
H25C	0.3195	0.6638	0.7709	0.073*	
C26	0.45504 (12)	0.6937 (2)	0.65493 (13)	0.0670 (7)	
H26A	0.4489	0.7400	0.6184	0.101*	
H26B	0.5052	0.6799	0.6626	0.101*	
H26C	0.4355	0.7295	0.6911	0.101*	
N1	0.23420 (8)	0.61223 (12)	0.49177 (7)	0.0379 (3)	
N2	0.14522 (10)	0.41316 (14)	0.71521 (9)	0.0547 (4)	
O1	0.10190 (8)	0.62053 (13)	0.49305 (8)	0.0582 (4)	
O2	0.16291 (9)	0.59563 (13)	0.39235 (7)	0.0590 (4)	
O3	0.20623 (10)	0.38349 (14)	0.70792 (11)	0.0838 (6)	
O4	0.09295 (11)	0.35658 (15)	0.70249 (11)	0.0870 (6)	
O5	0.41857 (6)	0.59176 (11)	0.64406 (6)	0.0428 (3)	
S1	0.15720 (3)	0.57206 (4)	0.45772 (2)	0.04322 (14)	
Cl1	0.08048 (3)	0.85490 (4)	0.81386 (3)	0.05761 (16)	
Cl2	0.00173 (3)	0.64492 (5)	0.86734 (2)	0.05700 (16)	

### Atomic displacement parameters (Å^2^)

**Table:**

	*U*^11^	*U*^22^	*U*^33^	*U*^12^	*U*^13^	*U*^23^
C1	0.0450 (10)	0.0334 (9)	0.0349 (9)	−0.0025 (7)	0.0085 (8)	0.0000 (7)
C2	0.0599 (13)	0.0586 (13)	0.0339 (10)	−0.0034 (10)	0.0054 (9)	0.0013 (8)
C3	0.0698 (15)	0.0659 (14)	0.0371 (11)	0.0007 (11)	0.0208 (10)	0.0041 (10)
C4	0.0507 (12)	0.0614 (13)	0.0508 (12)	0.0030 (9)	0.0243 (10)	0.0079 (10)
C5	0.0392 (10)	0.0472 (11)	0.0446 (11)	0.0025 (8)	0.0130 (8)	0.0073 (8)
C6	0.0398 (9)	0.0281 (8)	0.0340 (9)	−0.0001 (6)	0.0093 (7)	0.0018 (6)
C7	0.0335 (9)	0.0285 (8)	0.0345 (9)	−0.0007 (6)	0.0069 (7)	−0.0003 (6)
C8	0.0323 (9)	0.0323 (9)	0.0396 (9)	0.0011 (6)	0.0048 (7)	0.0018 (7)
C9	0.0375 (9)	0.0367 (9)	0.0341 (9)	0.0014 (7)	0.0053 (7)	0.0002 (7)
C10	0.0358 (9)	0.0333 (9)	0.0371 (9)	0.0002 (7)	0.0092 (7)	−0.0008 (7)
C11	0.0308 (9)	0.0411 (10)	0.0408 (10)	0.0002 (7)	0.0048 (7)	−0.0038 (7)
C12	0.0365 (9)	0.0314 (8)	0.0321 (9)	−0.0005 (6)	0.0031 (7)	−0.0017 (6)
C13	0.0347 (9)	0.0424 (10)	0.0333 (9)	0.0027 (7)	0.0056 (7)	0.0011 (7)
C14	0.0409 (10)	0.0411 (10)	0.0378 (10)	−0.0016 (7)	0.0112 (8)	0.0000 (7)
C15	0.0386 (9)	0.0455 (10)	0.0341 (9)	0.0034 (7)	0.0059 (7)	−0.0029 (7)
C16	0.0278 (8)	0.0575 (12)	0.0301 (9)	0.0015 (7)	0.0035 (7)	0.0003 (8)
C17	0.0334 (9)	0.0480 (11)	0.0411 (10)	−0.0046 (7)	0.0014 (8)	0.0074 (8)
C18	0.0361 (9)	0.0413 (10)	0.0391 (10)	0.0024 (7)	0.0045 (7)	0.0024 (7)
C19	0.0496 (12)	0.0459 (11)	0.0549 (12)	−0.0075 (8)	−0.0154 (10)	−0.0018 (9)
C20	0.0598 (14)	0.0521 (14)	0.0764 (16)	0.0053 (10)	−0.0227 (12)	−0.0114 (11)
C21	0.090 (2)	0.0527 (17)	0.134 (3)	0.0128 (14)	−0.053 (2)	−0.0278 (17)
C22	0.132 (3)	0.0504 (19)	0.155 (4)	−0.021 (2)	−0.068 (3)	0.013 (2)
C23	0.131 (3)	0.083 (3)	0.117 (3)	−0.054 (2)	−0.020 (3)	0.034 (2)
C24	0.091 (2)	0.0693 (17)	0.0765 (18)	−0.0285 (14)	−0.0045 (15)	0.0078 (13)
C25	0.0492 (12)	0.0635 (13)	0.0340 (10)	0.0033 (9)	0.0043 (9)	0.0026 (9)
C26	0.0422 (12)	0.0717 (16)	0.0870 (18)	−0.0142 (10)	−0.0034 (12)	0.0007 (13)
N1	0.0368 (8)	0.0445 (8)	0.0325 (8)	−0.0025 (6)	0.0025 (6)	−0.0024 (6)
N2	0.0576 (11)	0.0413 (10)	0.0659 (12)	−0.0001 (8)	0.0158 (9)	0.0014 (8)
O1	0.0407 (8)	0.0681 (10)	0.0655 (10)	0.0108 (7)	−0.0047 (7)	−0.0103 (7)
O2	0.0667 (10)	0.0666 (10)	0.0429 (8)	−0.0010 (7)	−0.0159 (7)	0.0057 (7)
O3	0.0677 (12)	0.0498 (10)	0.1355 (18)	0.0124 (8)	0.0353 (12)	−0.0033 (10)
O4	0.0775 (13)	0.0578 (11)	0.1262 (17)	−0.0173 (9)	0.0093 (12)	−0.0258 (10)
O5	0.0310 (6)	0.0498 (8)	0.0477 (8)	0.0013 (5)	0.0021 (6)	0.0047 (6)
S1	0.0422 (3)	0.0440 (3)	0.0430 (3)	0.00159 (18)	−0.0079 (2)	−0.00157 (19)
Cl1	0.0647 (4)	0.0520 (3)	0.0571 (3)	0.0080 (2)	0.0205 (3)	−0.0108 (2)
Cl2	0.0392 (3)	0.0869 (4)	0.0457 (3)	−0.0042 (2)	0.0174 (2)	−0.0040 (2)

### Geometric parameters (Å, º)

**Table:**

C1—C6	1.391 (3)	C16—C17	1.372 (3)
C1—C2	1.389 (3)	C16—Cl2	1.7144 (18)
C1—N1	1.426 (2)	C17—C18	1.379 (3)
C2—C3	1.373 (3)	C17—H17	0.9300
C2—H2	0.9300	C18—N2	1.462 (2)
C3—C4	1.373 (3)	C19—C24	1.362 (4)
C3—H3	0.9300	C19—C20	1.385 (3)
C4—C5	1.374 (3)	C19—S1	1.749 (2)
C4—H4	0.9300	C20—C21	1.382 (4)
C5—C6	1.389 (2)	C20—H20	0.9300
C5—H5	0.9300	C21—C22	1.361 (6)
C6—C7	1.444 (2)	C21—H21	0.9300
C7—C8	1.387 (2)	C22—C23	1.358 (6)
C7—C12	1.396 (2)	C22—H22	0.9300
C8—O5	1.377 (2)	C23—C24	1.382 (4)
C8—C9	1.390 (2)	C23—H23	0.9300
C9—C10	1.405 (2)	C24—H24	0.9300
C9—C25	1.503 (3)	C25—H25A	0.9600
C10—C11	1.382 (3)	C25—H25B	0.9600
C10—C13	1.492 (2)	C25—H25C	0.9600
C11—C12	1.379 (2)	C26—O5	1.430 (3)
C11—H11	0.9300	C26—H26A	0.9600
C12—N1	1.418 (2)	C26—H26B	0.9600
C13—C18	1.384 (3)	C26—H26C	0.9600
C13—C14	1.385 (3)	N1—S1	1.6623 (15)
C14—C15	1.380 (2)	N2—O3	1.207 (2)
C14—H14	0.9300	N2—O4	1.217 (2)
C15—C16	1.390 (3)	O1—S1	1.4163 (16)
C15—Cl1	1.7161 (19)	O2—S1	1.4173 (16)
			
C6—C1—C2	121.43 (18)	C16—C17—H17	120.4
C6—C1—N1	108.66 (15)	C18—C17—H17	120.4
C2—C1—N1	129.89 (18)	C17—C18—C13	123.32 (17)
C3—C2—C1	117.3 (2)	C17—C18—N2	116.71 (17)
C3—C2—H2	121.4	C13—C18—N2	119.95 (17)
C1—C2—H2	121.4	C24—C19—C20	120.8 (2)
C2—C3—C4	121.9 (2)	C24—C19—S1	120.0 (2)
C2—C3—H3	119.1	C20—C19—S1	119.23 (19)
C4—C3—H3	119.1	C21—C20—C19	118.8 (3)
C3—C4—C5	121.1 (2)	C21—C20—H20	120.6
C3—C4—H4	119.4	C19—C20—H20	120.6
C5—C4—H4	119.4	C22—C21—C20	120.2 (3)
C4—C5—C6	118.37 (19)	C22—C21—H21	119.9
C4—C5—H5	120.8	C20—C21—H21	119.9
C6—C5—H5	120.8	C21—C22—C23	120.6 (3)
C5—C6—C1	119.93 (17)	C21—C22—H22	119.7
C5—C6—C7	132.40 (17)	C23—C22—H22	119.7
C1—C6—C7	107.67 (15)	C22—C23—C24	120.4 (4)
C8—C7—C12	119.32 (15)	C22—C23—H23	119.8
C8—C7—C6	133.05 (16)	C24—C23—H23	119.8
C12—C7—C6	107.63 (15)	C19—C24—C23	119.3 (3)
O5—C8—C7	118.38 (15)	C19—C24—H24	120.4
O5—C8—C9	120.63 (16)	C23—C24—H24	120.4
C7—C8—C9	120.84 (16)	C9—C25—H25A	109.5
C8—C9—C10	117.74 (16)	C9—C25—H25B	109.5
C8—C9—C25	121.05 (16)	H25A—C25—H25B	109.5
C10—C9—C25	121.18 (16)	C9—C25—H25C	109.5
C11—C10—C9	122.69 (16)	H25A—C25—H25C	109.5
C11—C10—C13	116.92 (15)	H25B—C25—H25C	109.5
C9—C10—C13	120.32 (16)	O5—C26—H26A	109.5
C12—C11—C10	117.71 (16)	O5—C26—H26B	109.5
C12—C11—H11	121.1	H26A—C26—H26B	109.5
C10—C11—H11	121.1	O5—C26—H26C	109.5
C11—C12—C7	121.67 (16)	H26A—C26—H26C	109.5
C11—C12—N1	129.61 (16)	H26B—C26—H26C	109.5
C7—C12—N1	108.72 (15)	C12—N1—C1	107.23 (14)
C18—C13—C14	116.10 (16)	C12—N1—S1	122.47 (12)
C18—C13—C10	124.76 (16)	C1—N1—S1	124.15 (12)
C14—C13—C10	118.88 (16)	O3—N2—O4	123.7 (2)
C15—C14—C13	122.00 (17)	O3—N2—C18	118.82 (18)
C15—C14—H14	119.0	O4—N2—C18	117.53 (19)
C13—C14—H14	119.0	C8—O5—C26	114.36 (15)
C14—C15—C16	119.94 (17)	O2—S1—O1	120.21 (10)
C14—C15—Cl1	118.53 (14)	O2—S1—N1	106.07 (9)
C16—C15—Cl1	121.52 (14)	O1—S1—N1	106.33 (8)
C17—C16—C15	119.40 (17)	O2—S1—C19	109.16 (10)
C17—C16—Cl2	119.72 (15)	O1—S1—C19	108.80 (11)
C15—C16—Cl2	120.88 (15)	N1—S1—C19	105.23 (9)
C16—C17—C18	119.17 (17)		
			
C6—C1—C2—C3	0.5 (3)	C14—C15—C16—Cl2	176.85 (14)
N1—C1—C2—C3	178.70 (19)	Cl1—C15—C16—Cl2	−4.3 (2)
C1—C2—C3—C4	−1.2 (3)	C15—C16—C17—C18	2.2 (3)
C2—C3—C4—C5	0.9 (3)	Cl2—C16—C17—C18	−177.65 (13)
C3—C4—C5—C6	0.1 (3)	C16—C17—C18—C13	−0.2 (3)
C4—C5—C6—C1	−0.6 (3)	C16—C17—C18—N2	−178.70 (17)
C4—C5—C6—C7	179.07 (19)	C14—C13—C18—C17	−1.0 (3)
C2—C1—C6—C5	0.3 (3)	C10—C13—C18—C17	−175.02 (17)
N1—C1—C6—C5	−178.16 (15)	C14—C13—C18—N2	177.48 (17)
C2—C1—C6—C7	−179.44 (17)	C10—C13—C18—N2	3.4 (3)
N1—C1—C6—C7	2.06 (18)	C24—C19—C20—C21	0.1 (3)
C5—C6—C7—C8	−1.0 (3)	S1—C19—C20—C21	−178.99 (18)
C1—C6—C7—C8	178.70 (18)	C19—C20—C21—C22	0.3 (4)
C5—C6—C7—C12	−179.87 (18)	C20—C21—C22—C23	−0.8 (5)
C1—C6—C7—C12	−0.12 (18)	C21—C22—C23—C24	1.0 (5)
C12—C7—C8—O5	176.94 (14)	C20—C19—C24—C23	0.1 (4)
C6—C7—C8—O5	−1.8 (3)	S1—C19—C24—C23	179.2 (2)
C12—C7—C8—C9	1.3 (2)	C22—C23—C24—C19	−0.7 (5)
C6—C7—C8—C9	−177.38 (17)	C11—C12—N1—C1	−177.70 (17)
O5—C8—C9—C10	−176.46 (15)	C7—C12—N1—C1	3.11 (18)
C7—C8—C9—C10	−0.9 (2)	C11—C12—N1—S1	−24.3 (3)
O5—C8—C9—C25	1.6 (3)	C7—C12—N1—S1	156.49 (12)
C7—C8—C9—C25	177.13 (17)	C6—C1—N1—C12	−3.19 (18)
C8—C9—C10—C11	−0.6 (3)	C2—C1—N1—C12	178.47 (19)
C25—C9—C10—C11	−178.71 (17)	C6—C1—N1—S1	−156.01 (13)
C8—C9—C10—C13	−177.44 (16)	C2—C1—N1—S1	25.7 (3)
C25—C9—C10—C13	4.5 (3)	C17—C18—N2—O3	−138.2 (2)
C9—C10—C11—C12	1.8 (3)	C13—C18—N2—O3	43.3 (3)
C13—C10—C11—C12	178.68 (16)	C17—C18—N2—O4	41.6 (3)
C10—C11—C12—C7	−1.4 (3)	C13—C18—N2—O4	−136.9 (2)
C10—C11—C12—N1	179.54 (16)	C7—C8—O5—C26	96.3 (2)
C8—C7—C12—C11	−0.1 (2)	C9—C8—O5—C26	−88.1 (2)
C6—C7—C12—C11	178.87 (16)	C12—N1—S1—O2	175.29 (14)
C8—C7—C12—N1	179.12 (14)	C1—N1—S1—O2	−35.85 (17)
C6—C7—C12—N1	−1.87 (18)	C12—N1—S1—O1	46.24 (16)
C11—C10—C13—C18	82.6 (2)	C1—N1—S1—O1	−164.90 (15)
C9—C10—C13—C18	−100.5 (2)	C12—N1—S1—C19	−69.08 (16)
C11—C10—C13—C14	−91.3 (2)	C1—N1—S1—C19	79.77 (16)
C9—C10—C13—C14	85.6 (2)	C24—C19—S1—O2	−146.25 (19)
C18—C13—C14—C15	0.1 (3)	C20—C19—S1—O2	32.82 (19)
C10—C13—C14—C15	174.57 (16)	C24—C19—S1—O1	−13.3 (2)
C13—C14—C15—C16	1.8 (3)	C20—C19—S1—O1	165.73 (16)
C13—C14—C15—Cl1	−177.08 (14)	C24—C19—S1—N1	100.3 (2)
C14—C15—C16—C17	−3.0 (3)	C20—C19—S1—N1	−80.66 (17)
Cl1—C15—C16—C17	175.87 (14)		

### Hydrogen-bond geometry (Å, º)

**Table:**

*D*—H···*A*	*D*—H	H···*A*	*D*···*A*	*D*—H···*A*
C2—H2···O2	0.93	2.33	2.915 (3)	121
C11—H11···O1	0.93	2.36	2.955 (2)	122
